# Simultaneous induction of differentiation and senescence in cardiac fibroblasts by TGF-β1: effects of senotherapeutics drugs

**DOI:** 10.3389/fphar.2026.1877172

**Published:** 2026-08-14

**Authors:** Claudio Espinoza-Perez, José Miguel Osorio, Rubén Vélez, Juan Ortega Bustos, Víctor Machuca, Sebastián Rivas, Carlos F. Sánchez-Ferrer, Concepción Peiró, Raúl Vivar, Guillermo Díaz-Araya

**Affiliations:** 1 Department of Chemical Pharmacology and Toxicology, Faculty of Chemical and Pharmaceutical Sciences, University of Chile, Santiago, Chile; 2 Department of Pharmacology, Faculty of Medicine, Universidad Autónoma de Madrid, Madrid, Spain; 3 Instituto de Investigaciones Sanitarias IdiPAZ, Madrid, Spain; 4 Molecular and Clinical Pharmacology Program, Biomedical Sciences Institute, University of Chile, Santiago, Chile; 5 Advanced Center for Chronic Diseases, Faculty of Chemical and Pharmaceutical Sciences and Faculty of Medicine, University of Chile, Santiago, Chile

**Keywords:** cardiac myofibroblast, collagen, differentiation, senescence, TGF-β1

## Abstract

**Introduction:**

Aging is a major risk factor for cardiovascular diseases (CVD), partly due to the accumulation of senescent cells. Senescence is characterized by irreversible cell cycle arrest and the acquisition of a senescence associated secretory phenotype (SASP), which promotes inflammation and tissue remodeling, thereby contributing to cardiac dysfunction. Cardiac fibroblasts (CFs), key regulators of cardiac repair, differentiate into myofibroblasts (CMFs) in response to pathological stimuli such as mechanical stiffness and TGF-β1. CMFs secrete abundant extracellular matrix (ECM) proteins, and while their senescence may transiently restrict fibrosis in acute cardiac injury, persistent senescence promotes chronic remodeling through SASP activity. Given that TGF-β1 is a central mediator of CF-to-CMF differentiation and can induce senescence in various cell lines, we investigated whether it simultaneously triggers both processes in neonatal rat CFs, and whether the senotherapeutics Navitoclax and Dasatinib + Quercetin modulate the viability of senescent CMFs.

**Methods:**

CF from neonatal rats were incubated in DMEM-F12 (10% FBS), and treated with TGF-β1 (10 46 ng/mL, 7 days). Proteins were analyzed by Western blot and immunocytochemistry. Cytokines were analyzed by Milliplex. After 7 days cells were treated with senolytics drugs.

**Results:**

CFs treated with TGF-β1 exhibited senescence markers (p15-p16, p21), increased SA-β-gal activity, and reduced Ki-67 and p-Rb expression. CMFs displayed enhanced secretion of IL-1β, IL-6, IL-5, and IL-10, together with elevated collagen and VCAM-1 levels, indicating a pro-fibrotic and low-grade inflammatory phenotype. Treatment with Navitoclax or Dasatinib + Quercetin decreased cell viability and reduced the proportion of SA-β-gal -positive cells.

**Conclusion:**

TGF-β1 simultaneously promotes CF differentiation into CMFs and induces senescence, resulting in a sustained pro-fibrotic and inflammatory phenotype. Senotherapeutics treatment attenuates senescent CMFs, supporting its potential as a therapeutic strategy to mitigate cardiac fibrosis.

## 1 Introduction

Cellular senescence is defined as an irreversible arrest of the cell cycle accompanied by multiple alterations, including resistance to apoptosis, morphological changes, metabolic reprogramming, proteostasis disruption, increased oxidative stress, chromatin remodeling, and acquisition of a senescence-associated secretory phenotype (SASP). This secretory phenotype comprises cytokines, chemokines, metalloproteinases, and other bioactive factors (Herranz and Gil, 2018; Kirkland and Tchkonia, 2017). Both clinical and experimental evidence link senescence, senescent cell accumulation, and SASP release to cardiovascular diseases such as heart failure, myocardial infarction, arrhythmias, hypertension, atherosclerosis, and cardiac fibrosis (Kennedy et al., 2014; Niccoli and Partridge, 2012).

Senescence may be triggered by intrinsic factors (e.g., oxidative stress, telomere shortening, hyperproliferation) or extrinsic agents (e.g., UV light, γ-radiation, chemotherapeutic drugs), all converging on DNA damage (d'Adda di Fagagna, 2008). The DNA Damage Response (DDR), marked by γ-H2AX formation and activation of ATM and ATR kinases, initiates the p53/p21 pathway, while cell cycle arrest is further reinforced by the p16/Rb axis (Kumari and Jat, 2021). Morphologically, senescent cells are enlarged, flattened, and irregular, often showing vacuolization, multinucleation, and organelle alterations such as mitochondrial and ER enlargement. Increased lysosomal content underlies the characteristic rise in β-galactosidase activity (SA-β-gal) (Dimri et al., 1995; Huang et al., 2022).

Cardiac fibroblasts (CFs), which represent about 20% of cardiac cells, play essential structural and signaling roles through ECM maintenance and paracrine communication (Pinto et al., 2016; Humeres and Frangogiannis, 2019). Recently described as “sentinel cells”, CFs detect biochemical and mechanical changes in the myocardial microenvironment and adapt their behavior accordingly (Díaz-Araya et al., 2015). Our study does not aim to model natural organismal aging, but rather stress-induced premature senescence (SIPS) driven by a pro-fibrotic stimulus (TGF-β1). Neonatal fibroblasts provide a homogenous, highly reproducible, and unprimed baseline to study these direct target mechanisms without the confounding background of accumulated lifetime somatic mutations.

CFs senescence can be induced by stimuli such as angiotensin II, lipopolysaccharide, palmitate, and doxorubicin (Osorio et al., 2023). Their phenotype is dynamic: while transient senescence may restrict fibrosis during acute repair (e.g., post-infarction), persistent senescence promotes pathological remodeling through SASP activity, which activates neighboring fibroblasts and enhances ECM deposition, driving fibrotic progression (Chen et al., 2022).

A pivotal step in fibrosis is the differentiation of CFs into cardiac myofibroblasts (CMFs), characterized by α-SMA stress fiber assembly and robust ECM secretion (Kendall and Feghali-Bostwick, 2014; Turner and Porter, 2013). This transition is strongly influenced by mechanical stress (e.g., ECM stiffness), which promotes TGF-β1 secretion. TGF-β1 then acts in both autocrine and paracrine fashion to reinforce CFs-to-CMFs differentiation, while CMFs themselves secrete TGF-β1, perpetuating the fibrotic loop (Tomasek et al., 2002). Recognized as a master regulator of fibrosis, TGF-β1 is also capable of inducing senescence in multiple cell types, contributing to pathologies such as cardiovascular disease, Alzheimer’s disease, and obesity (Salvarani et al., 2017; Papageorgis, 2017). In fibroblasts and cancer cells, TGF-β1 exerts cytostatic effects through upregulation of CDK inhibitors (p15, p21, p27), leading to Rb hypophosphorylation and cell cycle arrest. In CFs, short-term TGF-β1 treatment increases p53/p21 expression and SA-β-gal activity (Li et al., 2019), while prolonged exposure (7 days) yields non-proliferative CMFs expressing high p15, reduced telomerase activity, and persistent ECM production (Petrov et al., 2008; Driesen et al., 2014). These findings suggest that TGF-β1-induced CMFs may acquire a senescent phenotype with pro-fibrotic SASP features, making them attractive therapeutic targets.

The chronic persistence of senescent cells is partly explained by activation of Senescent Cell Anti-apoptotic Pathways (SCAPs), including Bcl-2/Bcl-xL, p53/p21, PI3K/AKT, and Hsp90 signaling (Osorio et al., 2023; Soto-Gamez et al., 2019). This knowledge has fostered two therapeutic strategies: senolytics, which selectively eliminate senescent cells, and senomorphics (or senostatics), which suppress SASP secretion (Zhang et al., 2023). Both approaches have shown promise in improving healthspan and mitigating disease in preclinical models.

Thus, understanding the interplay between CFs senescence and CMFs differentiation under TGF-β1 stimulation opens new avenues for antifibrotic interventions. This study aims to characterize the senescence-associated changes in TGF-β1-induced CMFs and evaluate the potential of senotherapeutics drugs as modulators of this process.

## 2 Materials and methods

### 2.1 Materials

Organic and inorganic compounds (salts, acids, bases, and solvents), and paraformaldehyde were obtained from Merck (Darmstadt, Germany). Fetal Bovine Serum (FBS), rat tail collagen I, DMEM-F12 culture medium, trypan blue, collagenase type II, and trypsin-EDTA were acquired from Gibco (Carlsbad, CA, USA). RGB AccuRuler protein standard was sourced from Maestrogen (Hsinchu, Taiwan). ECL Clarity Max substrate, Bradford reagent, and bovine serum albumin (BSA) standard were purchased from Bio-Rad (Hercules, CA, USA). Bovine serum albumin–Fraction V was from Rockland (Philadelphia, PA, USA). Penicillin/streptomycin/amphotericin B solution and sterile plasticware for cell culture were obtained from Corning Inc. (New York, NY, USA). Senescence-associated β-galactosidase staining kit and protease/phosphatase inhibitor cocktail were sourced from Cell Signaling (Danvers, MA, USA). Doxorubicin, SB-431542, Triton X-100, and TGF-β1 were acquired from Sigma Aldrich (St. Louis, MO, USA). ProLong Gold Antifade with DAPI and Rhodamine Red dye was obtained from Invitrogen (Eugene, OR, USA). Primary antibodies: anti-p-Rb, Bax, Bcl-xL, γ-H2AX, and β-tubulin were purchased from Cell Signaling (Danvers, MA, USA); anti-p21, and α-SMA from Abcam (Cambridge, UK); and anti-p15-p16, from Santa Cruz Biotechnology Inc. (Dallas, TX, USA). Secondary antibodies conjugated to horseradish peroxidase (HRP) for anti-rabbit and anti-mouse, and secondary antibodies conjugated to Alexa Fluor® 488 nm were obtained from Cell Signaling (Danvers, MA, USA).

### 2.2 Isolation and culture of cardiac fibroblasts

Sprague-Dawley rats aged 2–3 days were employed. The animals were sourced from the animal facility of the Faculty of Chemical and Pharmaceutical Sciences. All procedures were conducted in accordance with the Guide for the Care and Use of Laboratory Animals, eighth Edition, 2011, published by the United States National Institutes of Health. For the isolation of neonatal rat CFs, the protocol established by the Laboratory of Molecular Signal Transduction (LTSM) was followed. Neonatal rats were decapitated, and their hearts were immediately excised. The hearts were finely minced and enzymatically digested using pancreatin and collagenase II. The resulting cell suspension was plated in 5% FBS for 2 h at 37 °C to promote CFs adhesion. After this period, the medium was removed and replaced with 10% FBS, allowing the cells to proliferate until reaching 70%–80% confluence. For passaging, CFs were detached using 0.1% trypsin in sterile 1X PBS, counted using the trypan blue exclusion method, and seeded into plastic culture plates in 10% FBS for 24 h, according to the experimental requirements. Subsequently, culture medium was refreshed 30 min prior to stimulation. In experiments without a deprivation phase, stimulation was performed by replacing the medium with fresh 10% FBS 30 min before adding the respective treatments. For collagen I secretion assay, all experimental groups received 100 µM ascorbic acid as an enzymatic cofactor essential for this process (see more in Section 2.8).

### 2.3 Experimental design

TGF-β1 treatment: CFs were treated with an initial pulse of TGF-β1 at 10 ng/mL (dissolved in sterile 1X PBS) for 7 days to assess its pro-senescent effect, as well as its well-documented pro-fibrotic activity. In none of these experiments the culture medium was changed during the treatment period. To experimentally prevent starvation and metabolic exhaustion over the 7-day period, cells were cultured using a high volume-to-surface area ratio of culture medium, heavily supplemented with 10% Fetal Bovine Serum (FBS).

Senotherapeutics treatment: After 7 days of treatment with TGF-β1, culture media was replaced with fresh medium previously to the treatments with navitoclax, dasatinib, quercetin or the combination of both. All drugs were dissolved in dimethyl sulfoxide (DMSO). Final concentration of DMSO in culture was 1%, and any potential solvent toxicity was considered in the vehicle group.

### 2.4 Senescence-associated β-galactosidase activity (SA-β-gal)

SA-β-gal staining was performed using the commercial kit from Cell Signaling Technology®, following the manufacturer’s instructions. CFs were seeded in 35 mm plates at density of 10^4^ cells per dish. After the treatment period, cells were washed three times with filtered, ice-cold 1X PBS. One milliliter of fixative solution (formaldehyde/glutaraldehyde) was added per plate for 10 min at room temperature, followed by three additional washes with filtered, ice-cold 1X PBS. Next, 1 mL of staining solution containing X-gal (1 mg/mL, pH 6.00 ± 0.01) was added to each plate and incubated for 24 h at 37 °C in a non-CO_2_ incubator. Following staining, plates were washed again with filtered, ice-cold 1X PBS, and each plate was divided into four quadrants for random image capture. Bright field images were taken using a Nikon Eclipse Ts2R microscope equipped with a Lanoptik MC4KW-G1 camera, using the Pixit Pro software. A minimum of 50 images per plate were captured, each containing at least 15 cells. For quantification, inclusion criteria required cells to have an area within the mean ±2 standard deviations and to exhibit blue perinuclear staining. At least 1,000 cells per plate were analyzed. Results were expressed as the percentage of positive cells per condition.

### 2.5 Immunofluorescence

Neonatal CFs were seeded onto coverslips placed in 35 mm plates at a density of 3 × 10^4^ cells per plate and treated with 10 ng/mL TGF-β1 for 7 days. After the experimental period, cells were washed three times with filtered, ice-cold 1X PBS, fixed with 4% paraformaldehyde (PFA) for 20 min at 4 °C, and then incubated in 100 mM glycine in 1X PBS for 15 min to reduce background caused by residual aldehydes from fixation. Cells were permeabilized with 0.1% Triton X-100 and blocked with 3% BSA in 1X PBS for 20 min to prevent non-specific binding. Following the blocking step, the primary antibody was incubated overnight at 4 °C in a humidified chamber. After incubation, cells were washed three times with 1X PBS and then incubated with the appropriate Alexa Fluor 488-conjugated secondary antibody (matching the isotype of the primary) for 2 h at room temperature. Coverslips were washed with 1X PBS and mounted onto microscope slides using ProLong Antifade Mountant with DAPI to stain the nuclei. Slides were stored at 4 °C until imaging. Fluorescence images were captured using an epifluorescence Nikon Eclipse Ts2R microscope equipped with a Lanoptik MC4KW-G1 camera and analyzed with Pixit Pro and ImageJ software (NIH, Bethesda, MD, USA). To quantify α-SMA fiber assembly, inclusion criteria required cells to have an area within the mean ±2 standard deviations and a clearly organized cytosolic stress fiber network. Primary and secondary antibodies used for immunofluorescence and their respective dilutions were: γ-H2AX 1:200/1:200 (anti-mouse), α-SMA 1:500/1:500 (anti-rabbit), Ki-67 1:200/1:200 (anti-rabbit), Rhodamine Red dye 1:500.

### 2.6 Cytokine determinations by multiplex assay

CFs supernatants were obtained by centrifuging at 21,380 × g for 10 min and stored at −80 °C prior to cytokine analysis. Before the procedure, samples were thawed and then analyzed for cytokine levels using the Milliplex™ MAP-Rat Cytokine Magnetic Bead Panel Immunology Multiplex Assay, according to the manufacturer’s instructions using a Luminex 200 System, Multiplex Bio-Assay. The cytokines measured included IL-1β, IL-6, IL-10, IL-5, MCP-1 and TNF-α. Standard curves were performed for each cytokine (1.5–10,000 pg/mL). Values were standardized as pg secreted cytokine/μg cell lysate protein.

### 2.7 Western blotting

After treatment, cells were washed with PBS, lysed with 50 μL of RIPA lysis buffer; the protein extract was sonicated and centrifuged (21,380 × g for 10 min) to remove cell debris. Protein concentration was assessed by the Bradford assay. Equivalent amounts of protein were separated by SDS-PAGE gels (8%–15% depending on the molecular weight of the protein of interest). Afterwards, proteins were transferred from the gels to PVDF membranes via electrotransfer at 0.1 A for 960 min. Membranes were then blocked for 60 min in either 5% skimmed milk powder or 5% bovine serum albumin (BSA) in 1X TBS with 0.1% (v/v) Tween-20 (TBS-T), considering whether or not the protein of interest exhibited phosphorylation. Membranes were incubated overnight at 4 °C with primary and secondary antibodies under constant agitation as indicated: p15-p16, 1:500; p21, 1:1000; p-Rb, 1:2000 VCAM-1, 1:1000; Bax, 1:1000; Bcl-xL, 1:1000; Bcl-2, 1:200; β-Tubulin, 1:1000; Secondary anti-mouse, 1:5000 and Secondary anti-rabbit, 1:5000. Protein detection was conducted via chemiluminescence using ECL substrate and visualized using the LICOR® c-Digit scanner model 3600 (Image Studio Digits v5.2 software). Image analysis was carried out with the software’s quantification tool, and relative expression levels were normalized against β-tubulin as loading control.

### 2.8 Measurement of secreted collagen

After stimulation with TGF-β1, culture media was changed for fresh medium, and 100 µM ascorbic acid was added as enzymatic cofactor of collagen synthesis in every experimental group. After 48 h of incubation, the culture medium was collected and centrifuged at 21,380 × g for 10 min to remove cell debris. The medium was incubated for 24 h at 4 °C with constant agitation in 25% (w/v) ammonium sulphate ((NH_4_)_2_SO_4_) aqueous solution, at a 1:3 ratio (1 part medium to 3 parts ammonium sulphate solution) to precipitate proteins. Following incubation, samples were centrifuged at 6,000 × g for 60 min to collect the precipitated protein pellet. The pellet was dissolved in 1 mL of 0.5 M acetic acid. Aliquots of 100 µL were incubated with 950 µL of 50 µM Sirius Red (69 μg/mL) for 30 min at room temperature. After staining, samples were centrifuged at 21,380 × g for 30 min at 4 °C to pellet the red-stained collagen fibres. Excess Sirius Red solution was discarded, and the pellet was incubated in 0.1 M KOH for 15 min to elute the dye. Absorbance was measured in triplicate at 590 nm, and values were interpolated from a standard curve prepared with rat tail collagen I at concentrations of 0, 10, 25, 50, 100, 200, 300, and 400 μg/mL. The collagen quantity in each sample was calculated by multiplying the interpolated value by the resuspension volume (1 mL), and normalized to the total protein content in the corresponding cell lysates. Results were expressed as µg of collagen per µg of cell lysate protein.

### 2.9 Statistical analyses

All data are presented as mean ± SD from 3 to 8 independent experiments. First, normality and homogeneity of variances were assessed for each variable using the Shapiro–Wilk and Brown–Forsythe tests, respectively. An unpaired two-tailed Student’s t-test (for parametric variables) or the Mann–Whitney test (for non-parametric variables) was used to determine differences between two groups. One-way analysis of variance (ANOVA), followed by Tukey’s *post hoc* test (for parametric variables) or the Kruskal–Wallis test followed by Dunn’s multiple-comparison *post hoc* test (for non-parametric variables), was applied to evaluate differences among three or more groups. For experimental designs with grouped variables, two-way ANOVA followed by Tukey’s multiple-comparison test was used. The statistical test applied to each dataset is indicated in the figure legends. Statistical significance was defined as *p* ≤ 0.05. All analyses were performed using GraphPad Prism 9.0.2 (California, USA).

## 3 Results

### 3.1 TGF-β1 induces differentiation of cardiac fibroblasts into myofibroblasts

**FIGURE 1:**
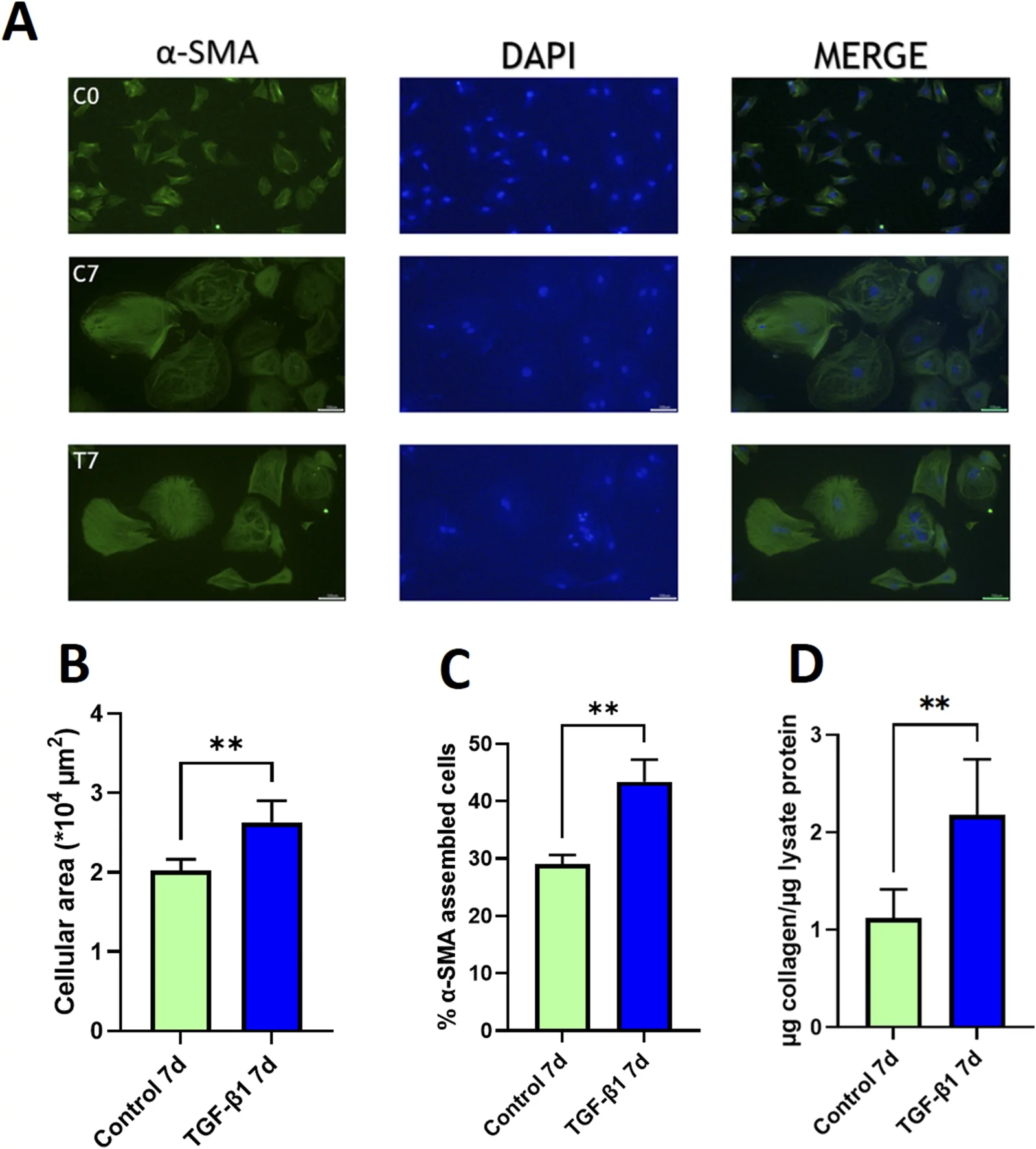
TGF-β1 induces the differentiation of cardiac fibroblasts (CF) into cardiac myofibroblasts (CMF). CFs were treated with TGF-β1 (10 ng/ml) or vehicle (1X PBS) for 7 days. Immunofluorescence for α-SMA (green) and nuclei with DAPI (blue) was performed; representative images at 10× (scale bar 100 µm) are shown to evaluate α-SMA assembly into stress fibers **(A)**, percentage of cells with α-SMA assembly **(B)**, cells with increased cellular area **(C)** (N = 5). To measure soluble collagen **(D)**, after 7 days of TGF-β1 treatment, cells were further incubated for 48 h in fresh medium with ascorbic acid (100 µM), and soluble collagen was quantified by Sirius Red staining and spectrophotometry, expressed as µg collagen/µg protein (N = 7). Control vs TGF-β1 were compared using unpaired Mann -Whitney; ** p < 0.01.

The expression and organization of α-SMA into stress fibers were assessed by immunofluorescence, and cell area was measured since CMFs are typically larger than CFs. As shown in Figures 1A,B, CFs treated with TGF-β1 for 7 days displayed stronger green fluorescence, indicative of increased α-SMA expression, compared to untreated controls. A reference group (Control 0; fibroblasts cultured ≤4 days) was included to illustrate culture-related morphological changes, showing smaller cells with weak, diffuse α-SMA staining. By contrast, TGF-β1 stimulation markedly increased the proportion of cells with well-structured α-SMA stress fibers compared to day 7 controls, in which staining remained diffuse. Consistently, TGF-β1-treated cells displayed a significantly larger surface area (Figure 1C). These findings confirm the differentiation of CFs into CMFs under TGF-β1 stimulation. To further validate the CMF phenotype, collagen secretion into the culture medium was quantified using Sirius Red precipitation and spectrophotometric analysis. As shown in Figure 1D, TGF-β1 treatment significantly increased collagen secretion, supporting the acquisition of a pro-fibrotic phenotype characterized by elevated ECM production.

### 3.2 TGF-β1 induces senescence-associated protein expression

**FIGURE 2:**
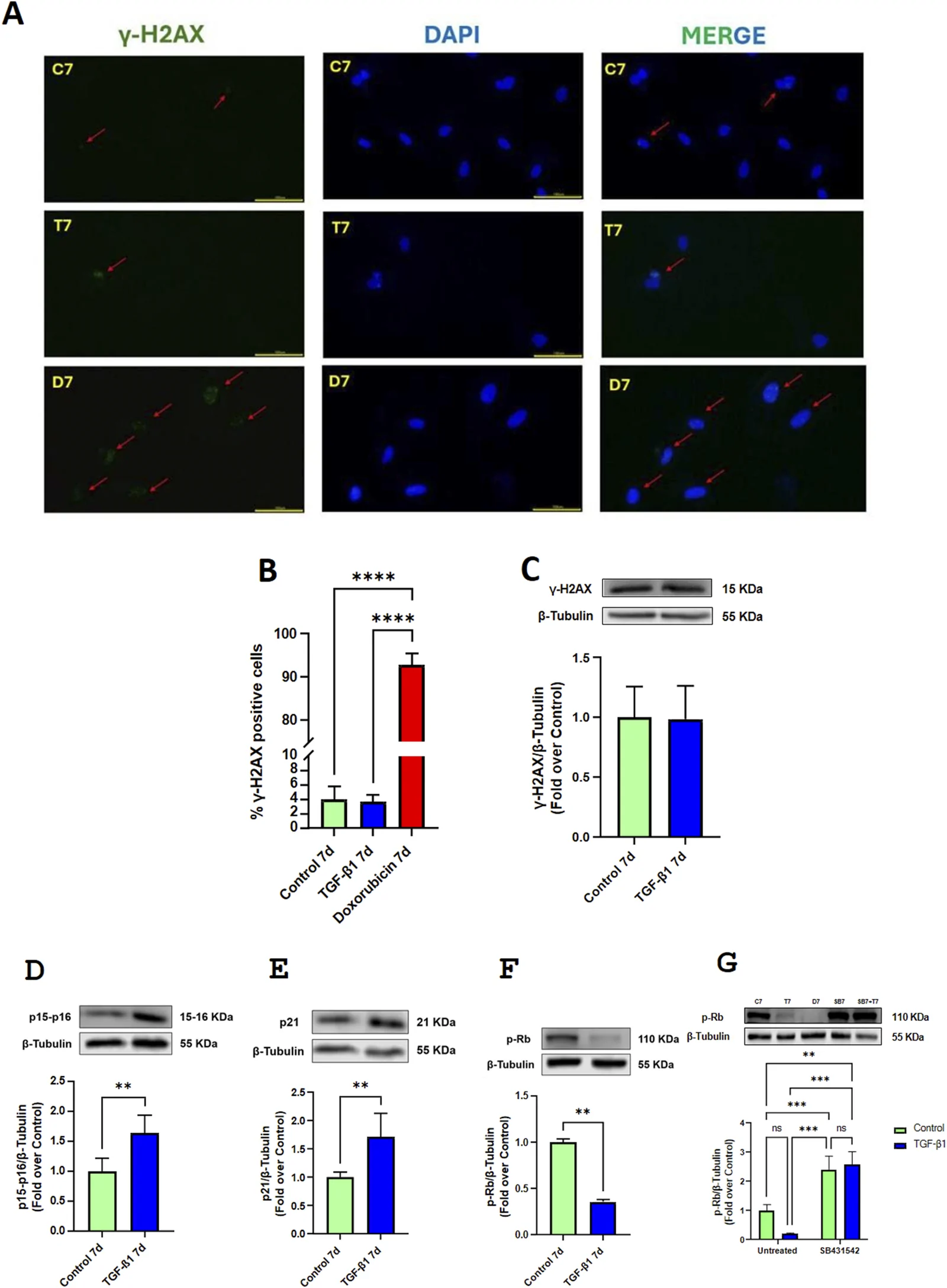
TGF-β1 alters cell cycle proteins without inducing DNA damage. CFs were treated with TGF-β1 (10 ng/mL) for 7 days or vehicle (1X PBS). Immunofluorescence **(A,B)** and Western blot **(C)** were performed for γ-H2AX (green; nuclei counterstained with DAPI, blue). Representative images taken with epifluorescence microscopy at ×20 magnification, indicated at the bottom right corner, where the scale bar denotes 100 μm. p15–p16 **(D)** p21 **(E)** and Rb phosphorylation **(F,G)** were analyzed by Western blot, with β-Tubulin as a loading control. In **(G)** the ALK5 inhibitor SB431542 (10 μM, 7 days) was included. N = 5 for graphs **(A–F)** N = 3 for **(G)**. Control vs. TGF-β1 comparisons were performed by unpaired Mann–Whitney; p-Rb with SB431542 was analyzed by two-way ANOVA followed by Tukey’s multiple comparisons test. ***p < 0.001, **p < 0.01. D7 (doxorubicin 100 nM, 7 days) was included as a positive control.

To assess whether TGF-β1 induces DNA damage, γ-H2AX levels were evaluated after 7 days of treatment. Immunofluorescence analysis showed no significant changes in γ-H2AX positivity between control and TGF-β1 groups (Figures 2A,B), whereas nearly all doxorubicin-treated cells (as positive control) exhibited nuclear γ-H2AX staining, as expected. Western blot analysis corroborated the absence of γ-H2AX upregulation in TGF-β1-treated cells (Figure 2C). In contrast, TGF-β1 markedly increased the expression of CDK inhibitors p15/p16 (Figure 2D) and p21 (Figure 2E), while reducing Rb phosphorylation (Figure 2F), compared to controls. These results indicate the induction of senescence-associated signaling. Importantly, these effects were observed despite culture in 10% FBS, a strongly pro-mitogenic condition, further supporting the loss of proliferative capacity in TGF-β1-induced CMFs. To confirm receptor involvement, the ALK5 inhibitor SB431542 (10 µM) was applied. As shown in Figure 2G, SB431542 reversed the TGF-β1-induced reduction in pRb phosphorylation, confirming dependence on TβRI/TβRII signaling. Interestingly, the group treated with SB431542 for 7 days exhibited higher levels of Rb phosphorylation than the untreated control, suggesting that autocrine/paracrine TGF-β1 secretion triggered by mechanical stress may progressively suppress proliferation in long-term cell cultures.

### 3.3 TGF-β1 reduces proliferation and increases SA-β-gal activity

**FIGURE 3:**
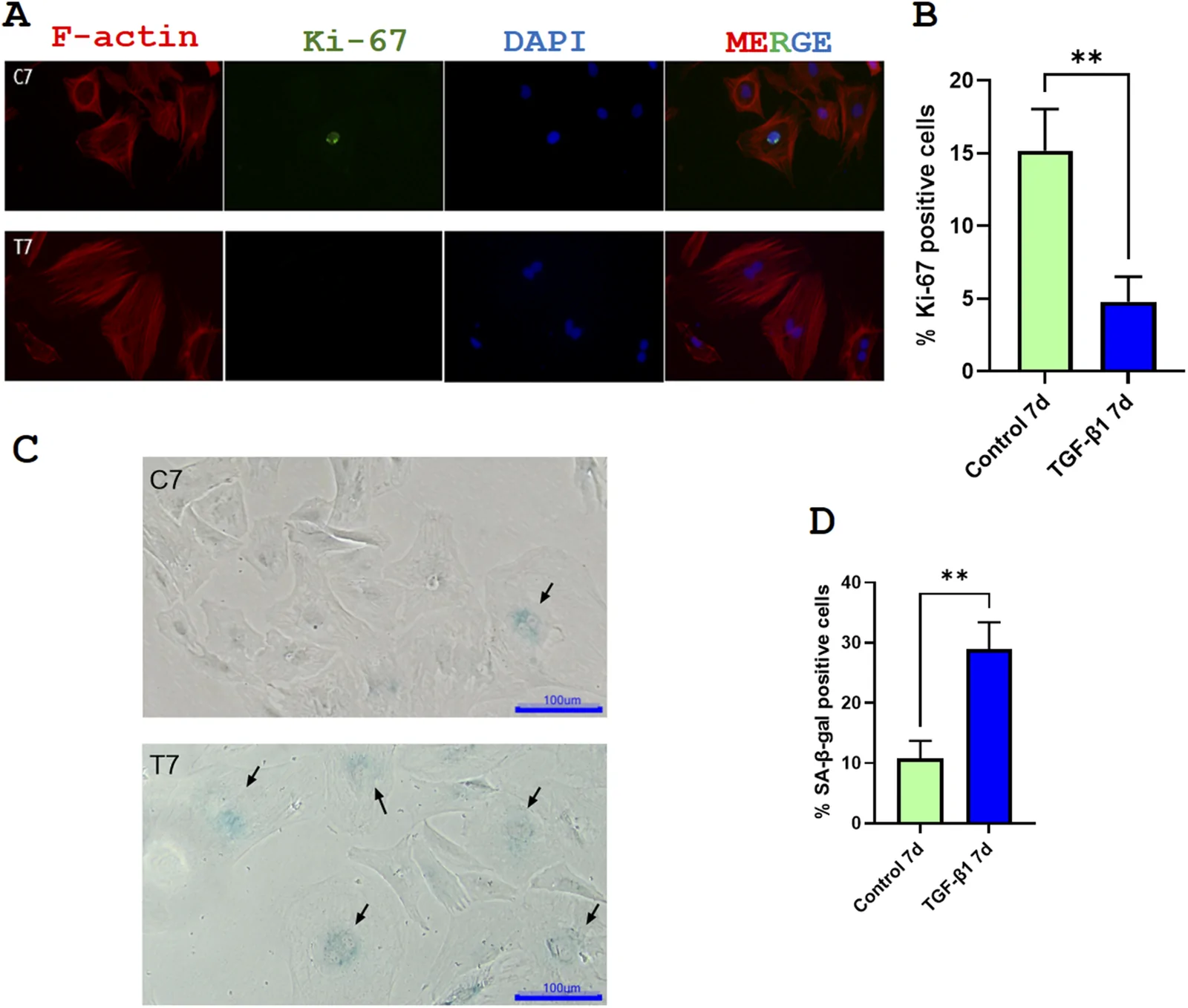
TGF-β1 reduces proliferation and increases SA-β-gal staining. CFs were treated with TGF-β1 (10 ng/ml) or vehicle (1X PBS) for 7 days. After treatment, immunofluorescence for Ki-67 (green; **(A, B)**) was performed. Nuclei were stained with DAPI (blue) and the cytoskeleton (F-actin) with rhodamine (red). Representative images were acquired using epifluorescence microscopy at 20× magnification, indicated at the bottom right, with a scale bar of 100 µm. Additionally, SA-β-gal staining was performed at pH 6 **(C, D)**. Representative images were acquired by brightfield microscopy at 10× magnification, indicated at the bottom right, with a scale bar of 100 µm. C7 = Control 7 days; T7 = TGF-β1 7 days. N = 5. In both graphs, Control vs TGF-β1 groups were compared by unpaired non-parametric Mann -Whitney test. **p < 0.01.

Proliferation was assessed by Ki-67 immunofluorescence. As shown in Figures 3A,B, TGF-β1-treated cells exhibited a significantly lower proportion of Ki-67 positivity compared with controls, consistent with proliferative arrest. In parallel, SA-β-Gal staining revealed a significant increase in positive cells in the TGF-β1 group versus controls at day 7 (Figures 3C,D). Together, these findings confirm the induction of senescence.

### 3.4 TGF-β1 induces cytokine secretion (SASP) in senescent CMFs

**FIGURE 4:**
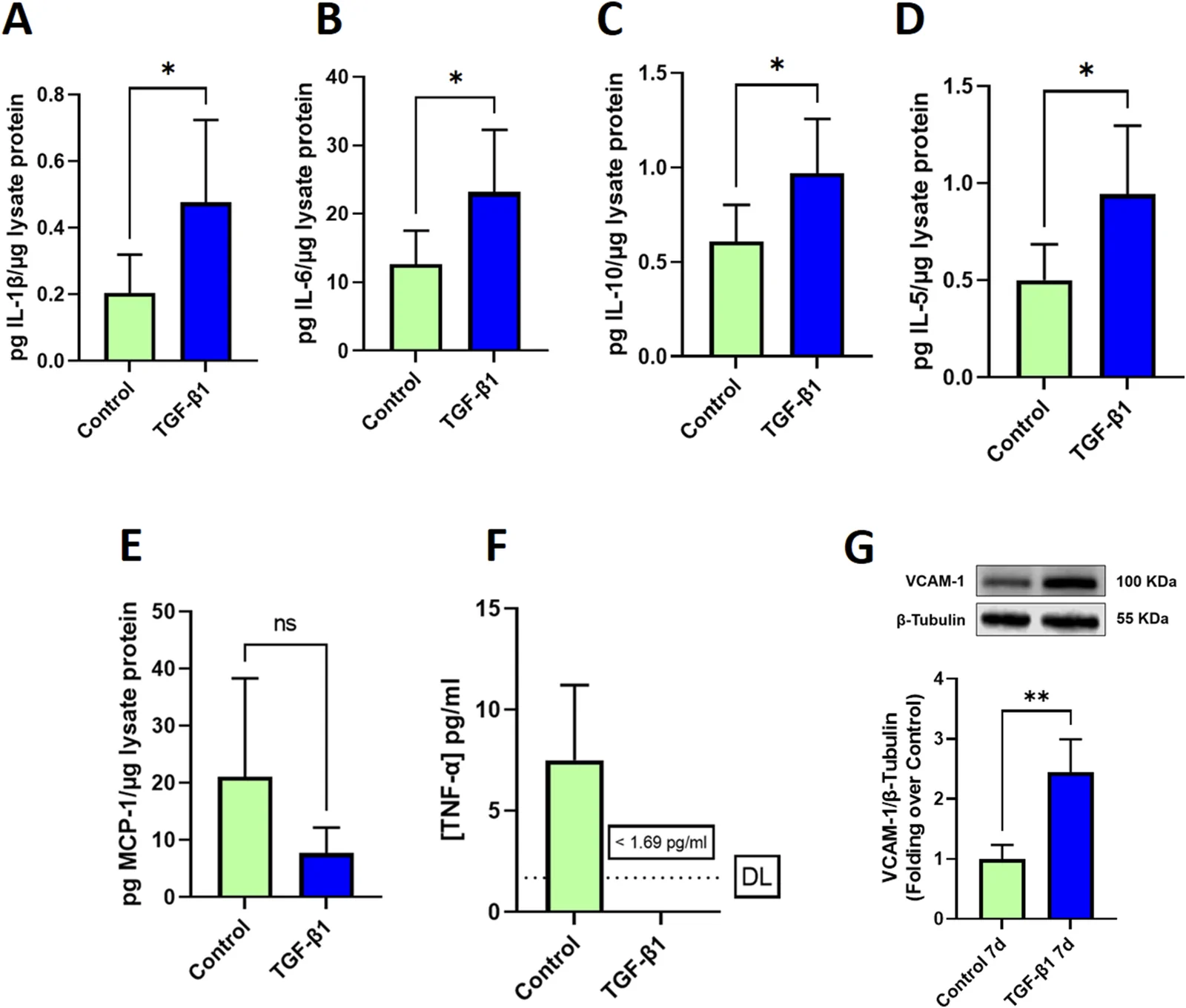
SASP profile of senescent myofibroblasts induced by TGF-β1. CFs were treated with TGF-β1 (10 ng/mL) or vehicle (1X PBS) for 7 days. After this period, cells were incubated for 48 h in DMEM-F12 medium, and secreted cytokines were measured by Luminex: IL-1β **(A)** IL-6 **(B)** IL-10 **(C)** IL-5 **(D)** MCP-1 **(E)** and TNF-α [**(F)** pg/mL], expressed as pg/µg protein of cell lysate except for TNF-α; N = 8. Except for TNF-α, Control vs. TGF-β1 was compared by Mann–Whitney; *p < 0.05, ns: not significant. VCAM-1 **(G)** was analyzed by Western blot (β-Tubulin as loading control; N = 5); Control vs. TGF-β1, Mann–Whitney, **p < 0.01. LD: limit of detection.

We next evaluated SASP-associated cytokines and chemokines, as well as the adhesion molecule VCAM-1. As shown in Figures 4A–G, TGF-β1-treated CMFs secreted higher levels of IL-1β, IL-6, IL-10, and IL-5, while MCP-1 displayed a non-significant reduction due to variability. TNF-α was undetectable in the TGF-β1 group (<1.69 pg/mL). In addition, VCAM-1 levels were significantly elevated. These results indicate that TGF-β1-induced senescent CMFs adopt a mixed SASP, with both pro-inflammatory (IL-1β, IL-6, VCAM-1) and anti-inflammatory (IL-5, IL-10) components, but low TNF-α secretion.

### 3.5 TGF-β1 modulates pro- and anti-apoptotic protein expression

**FIGURE 5:**
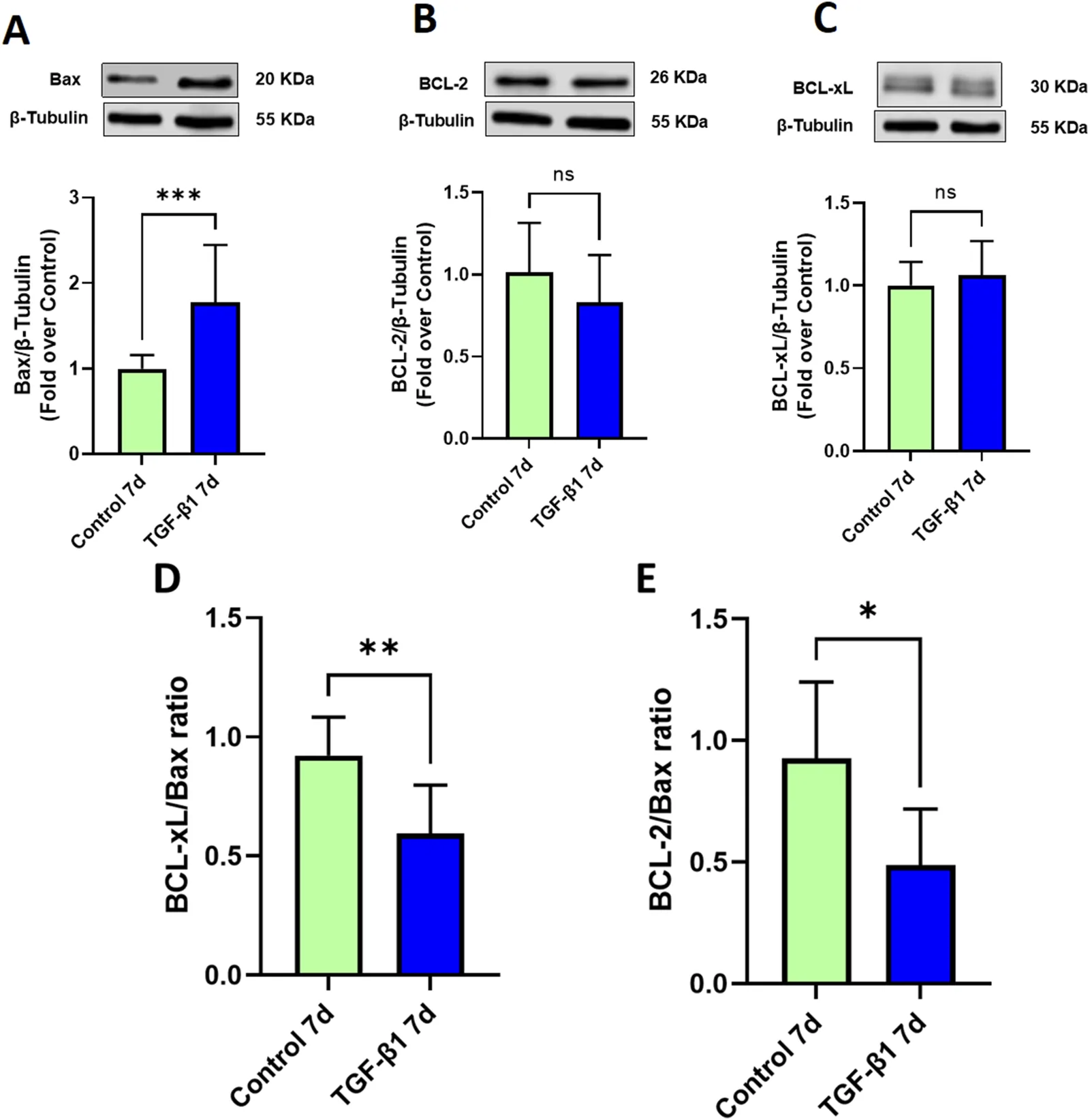
TGF-β1 increases Bax levels. CFs were treated with TGF-β1 (10 ng/mL) for 7 days or vehicle (1X PBS). Bax **(A)** BCL-2 **(B)** and BCL-xL **(C)** levels were analyzed by Western blot, with β-Tubulin as a loading control. Ratios of BCL-xL/Bax **(D)** and BCL-2/Bax **(E)** were also calculated to assess the apoptotic state of the cells. Control vs. TGF-β1 comparisons were performed using unpaired Mann–Whitney; *p < 0.05, ***p < 0.001.

Western blot analysis revealed increased Bax levels in TGF-β1-treated cells (Figure 5A), while Bcl-2 (Figure 5B) and Bcl-XL (Figure 5C) remained unchanged. Calculation of apoptotic ratios showed a significant reduction in both Bcl-XL/Bax (Figure 5D) and Bcl-2/Bax (Figure 5E), indicating a shift toward pro-apoptotic signaling due to increased Bax expression.

### 3.6 Senotherapeutics drugs reduce viability and senescence of CMFs

**FIGURE 6:**
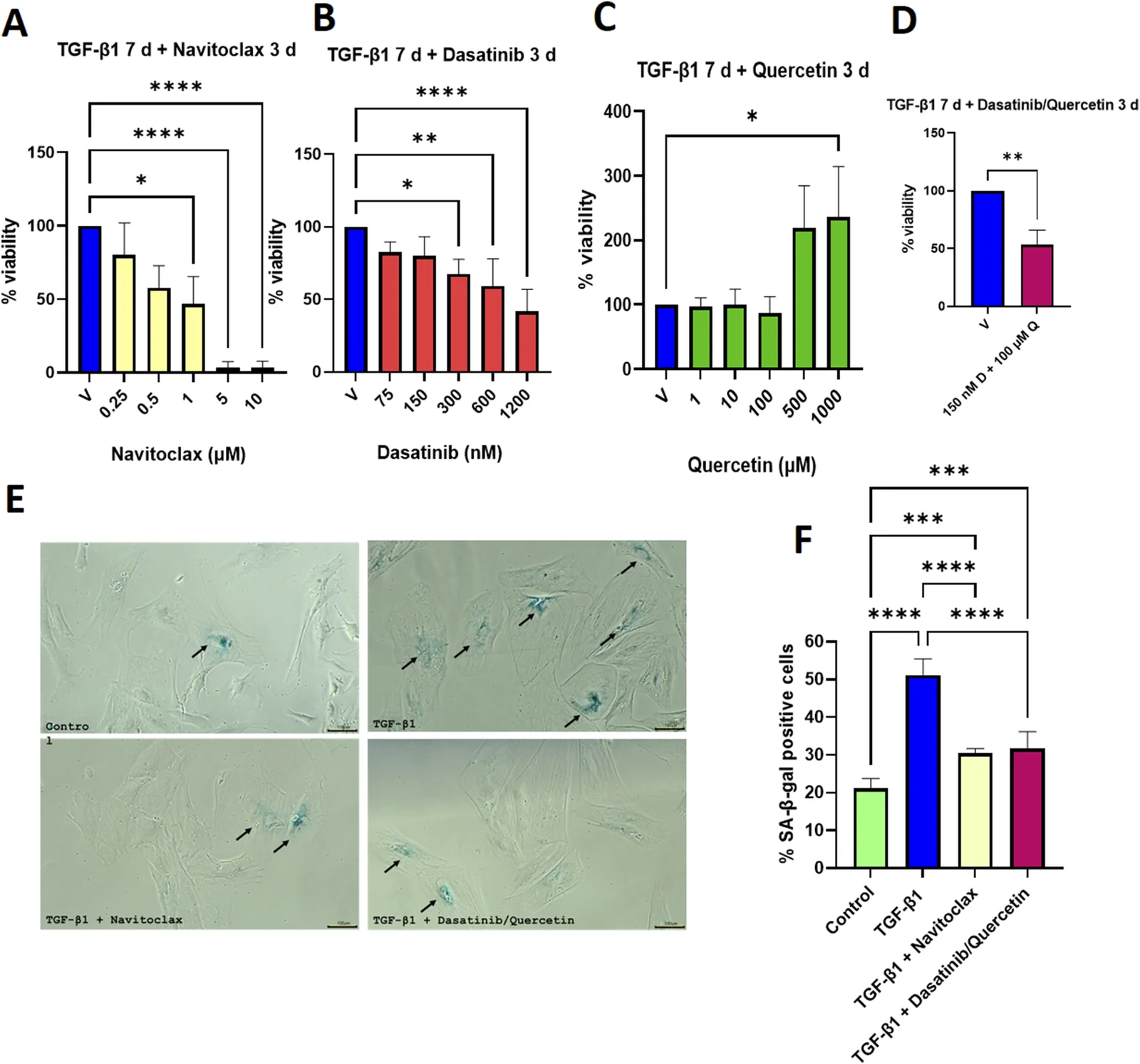
Senolytic drugs reduce viability and SA-β-gal staining of senescent CMF. After treatment with TGF-β1 (10 ng/mL) for 7 days, medium was replaced and cells were exposed to increasing doses of Navitoclax **(A)** Dasatinib **(B)** Quercetin **(C)** or a fixed dose of Dasatinib + Quercetin **(D)**. Cell viability was assessed by MTT after 72 h of drug treatment. Additionally, SA-β-gal staining **(E,F)** was performed in senescent CMF treated with Navitoclax (1 µM) or the combination of Dasatinib (150 nM) + Quercetin (100 µM). For figures (A, N = 8) and (B–C, N = 6), Vehicle (V, 1% DMSO) was compared against drug-treated groups using one-way ANOVA followed by Dunnett´s multiple comparisons pos-hoc test; (D, N = 6) was analyzed by unpaired Mann–Whitney; (E, N = 6) was analyzed by one-way ANOVA followed by Šídák’s test. *p < 0.05, **p < 0.01, ***p < 0.001, ****p < 0.0001.

The effects of senotherapeuticss were next examined. Navitoclax treatment for 3 days significantly reduced viability of TGF-β1-induced CMFs in a dose-dependent manner (Figure 6A), with effects evident at ≥ 1 µM. Based on these findings, 1 µM was selected for subsequent experiments. Dasatinib, Quercetin, and their combination showed compound- and dose-dependent effects (Figures 6B–D). Dasatinib (300–1200 nM) reduced viability in a dose-dependent fashion, while Quercetin produced divergent outcomes: lower doses (1–100 µM) had no effect in viability, but higher doses (500–1000 µM) paradoxically increased viability, reaching significance at the highest concentrations. Importantly, the combination of Dasatinib (150 nM) and Quercetin (100 µM) reduced viability by ∼50% compared with vehicle-treated controls. Consistent with these findings, SA-β-Gal activity analysis (Figures 6E,F) showed that TGF-β1 treatment increased the proportion of senescent cells (∼50% vs. ∼20% in controls). Navitoclax and Dasatinib + Quercetin both reduced SA-β-Gal positivity to ∼30%, representing significant decreases compared to untreated senescent cells.

## 4 Discussion

In the heart, cardiac fibrosis is associated with the progressive accumulation of senescent fibroblasts and myofibroblasts. Far from being inert, these cells secrete a repertoire of inflammatory and fibrogenic mediators collectively known as SASP. This secretome amplifies fibrosis by activating neighboring fibroblasts and promoting maladaptive tissue remodeling, ultimately impairing cardiac function and contributing to the progression toward heart failure. The relevance of SASP as a driver of inflammation and fibrosis has stimulated the development of senolytic and senostatic drugs. However, their translation to the clinic has faced challenges, including adverse effects and variability in efficacy.

### 4.1 Differentiation and TGF-β1–induced senescence

Exposure of CFs to TGF-β1 promotes their differentiation into CMFs, a process characterized by α-SMA expression and its assembly into stress fibers, enhancing contractile capacity and collagen secretion ((Petrov et al., 2008; Petrov et al., 2002)). Our results confirm this effect through both α-SMA organization and increased contractility. Simultaneously, TGF-β1 stimulation induced a senescent phenotype, as indicated by upregulation of p15^Ink4b^, p16^Ink4a^, and p21, hypophosphorylation of Rb, reduced proliferation (Ki-67), and increased SA-β-gal activity, in the absence of detectable DNA damage (γ-H2AX).

These findings suggest that TGF-β1–induced differentiation and senescence are tightly linked. Previous studies described transient p21 activation under TGF-β1 treatment (Datto et al., 1995; Reynisdóttir et al., 1995), but our data reveal sustained activation, indicating long-term cell cycle arrest in CMFs. This outcome is consistent with other models where TGF-β1 signaling induces premature senescence through p15/p16 axis (Senturk et al., 2010).

The convergence of differentiation and senescence may have functional implications. During repair, CFs proliferate and differentiate into CMFs to increase the deposit of extracellular matrix (ECM), but once an activation threshold is reached, senescence could act as a brake to limit proliferation and prevent uncontrolled fibrosis, a phenomenon also observed in other pathological models such as liver fibrosis (Krizhanovsky et al., 2008). Nevertheless, persistence of senescent CMFs with an altered secretory phenotype may instead promote chronic and fibrotic cardiac remodeling in the long term.

### 4.2 Secretory profile of senescent CMFs

TGF-β1–induced senescence altered not only proliferative capacity but also the secretory profile of CMFs. We found increased secretion of IL-1β, IL-6, IL-5, and IL-10, together with enhanced collagen and VCAM-1 expression, whereas TNF-α secretion was diminished. This pattern corresponds to a “low-grade SASP” combining pro- and anti-inflammatory features, with a pronounced pro-fibrotic bias. These results align partially with previous reports. Li et al. (Li et al., 2023) showed TGF-β1–induced fibroblast senescence enriches IL-6/IL-8, while Petrov et al. (Petrov et al., 2008; Petrov et al., 2002), established that TGF-β1 induces growth arrest, senescence markers and enhanced collagen synthesis in CF. However, our study extends these findings by combining a comprehensive cytokine profiling of this specific senescent population with a pharmacological evaluation of senotherapeutic vulnerabilities (such as the effects of senolytics). Our novelty lies in showing collagen secretion within a senescent context, suggesting senescent CMFs remain active players in ECM remodeling. The reduction in TNF-α is particularly noteworthy, as this cytokine often sustains chronic inflammation. Its decrease suggests a distinct SASP modulation in CMFs, possibly reflecting the interplay between TGF-β1 signaling and anti-inflammatory pathways. Moreover, matrix stiffness and mechanical stress can regulate SASP heterogeneity (Mebratu et al., 2023), an aspect worth exploring in future models using physiologically and pathologically relevant ECM stiffness.

The helper senescent cells (which are “transient/physiological senescent cells, or cells exerting acute, beneficial paracrine signaling) vs. harmful senescent cells (represent “chronic/pathological senescent cells”) framework (Kirkland and Tchkonia, 2020) helps interpret these findings: senescent CMFs may initially restrain fibrosis and modulate inflammation being helper senescence cells, but their persistence, coupled with collagen secretion and chronic SASP activity, likely accelerates pathological remodeling being detrimental senescence cells.

### 4.3 TGF-β1 promotes a proapoptotic profile in neonatal rat CMFs

TGF-β1–induced senescence in CFs was associated with a distinct alteration in the expression of Bcl-2 family proteins. Specifically, Bax levels significantly increased, while the expression of the anti-apoptotic proteins Bcl-2 and Bcl-XL remained unchanged. This differential regulation shifted both the Bcl-2/Bax and Bcl-XL/Bax ratios toward a pro-apoptotic state.

Classically, Bax promotes mitochondrial outer membrane permeabilization to trigger the apoptotic cascade, a process directly counteracted by Bcl-2 and Bcl-XL (Youle and Strasser, 2008). In our system, the persistence and survival of senescent CMFs despite elevated Bax levels suggests that stable baseline expression of Bcl-2/Bcl-XL is sufficient to keep these cells alive. This phenotype aligns with the concept of 'mitochondrial apoptosis priming', where senescent cells maintain a precarious survival balance under chronic apoptotic pressure, depending heavily on their remaining anti-apoptotic capacity to sequester excess pro-apoptotic signals.

### 4.4 Effects of senotherapeutics drugs on senescent CMFs

Our results demonstrate that TGF-β1–induced senescent CMFs are sensitive to Navitoclax and Dasatinib + Quercetin, confirming previous reports of their senotherapeutics efficacy across cell types (Kirkland and Tchkonia, 2020; Zhu et al., 2016). Navitoclax induced dose-dependent reductions in viability at ≥ 1 µM and decreased SA-β-gal positivity, consistent with its role as a Bcl-2/Bcl-XL inhibitor.

Interestingly, despite no increase in Bcl-2/Bcl-XL levels in our system, Navitoclax effectively reduced senescent CMF viability. This shifted molecular balance—characterized by increased Bax with stable baseline anti-apoptotic proteins—creates a specific, druggable vulnerability. Under the framework of 'mitochondrial apoptosis priming', these cells likely maintain a precarious survival balance under chronic apoptotic pressure, depending heavily on baseline Bcl-2/Bcl-XL levels to sequester the excess pro-apoptotic Bax. When exposed to Navitoclax, this delicate homeostatic buffer is disrupted, presumably unleashing the accumulated Bax pool to execute selective apoptosis.

Similarly, the combination of Dasatinib and Quercetin (D + Q), which targets broader cell survival networks (SCAPs), successfully reduced senescent CMF viability (∼50%) and markedly decreased senescence markers, confirming synergistic senotherapeutic activity (Raffaele et al., 2021). Dasatinib alone reduced viability in the nanomolar range, while Quercetin showed a paradoxical pro-viability effect at high concentrations, consistent with reported pleiotropic antioxidant actions. Taken together, these findings confirm that senescent CMFs depend on active anti-apoptotic signaling pathways for their persistence.

While our results strongly suggest a mechanism driven by mitochondrial priming, a limitation of the current study is the absence of functional cell death assays (such as Annexin V/PI flow cytometry or caspase-3/7 activation kinetics), to definitively track the execution of apoptosis. Future investigations will be essential to precisely characterize the mitochondrial dependence of these senescent populations.

Consequently, these findings position senolytics as promising experimental tools to selectively target senescent CMFs in fibrotic environments. Yet, translational challenges remain: Navitoclax is associated with thrombocytopenia and neutropenia, while Dasatinib + Quercetin efficacy varies by context. Exploring targeted delivery strategies (e.g., nanoparticles) or combining senolytics with conventional antifibrotics (e.g., RAAS inhibitors, pirfenidone) may enhance therapeutic potential while limiting toxicity. Therefore, senolytics must be viewed strictly as potential pharmacological tools whose *in vivo* efficacy, safety profile, and delivery methods require extensive future validation.

### 4.5 Perspectives, translational implications and limitations

Our results highlight senescent CMFs as active contributors to pathological remodeling rather than passive bystanders. The data support senolytic strategies as potential antifibrotic interventions. Nevertheless, further studies are needed to: Assess efficacy in adult CFs and *in vivo* cardiac fibrosis models, as neonatal cells may not fully recapitulate adult responses. Explore interactions between senescent CMFs and cardiomyocytes or immune cells, since paracrine crosstalk is central to remodeling. Investigate mechanical microenvironment effects on SASP modulation and senolytic sensitivity. Optimize dosing, timing, and safety profiles of senolytic interventions, possibly integrating senostatics (e.g., metformin, rapamycin) to complement clearance strategies are all attractive subjects for further research. The main limitations is the use of neonatal cardiac fibroblast and these *in vitro* findings need to be further study in aged *in vivo* hearts.

### 4.6 Conclusion

**FIGURE 7:**
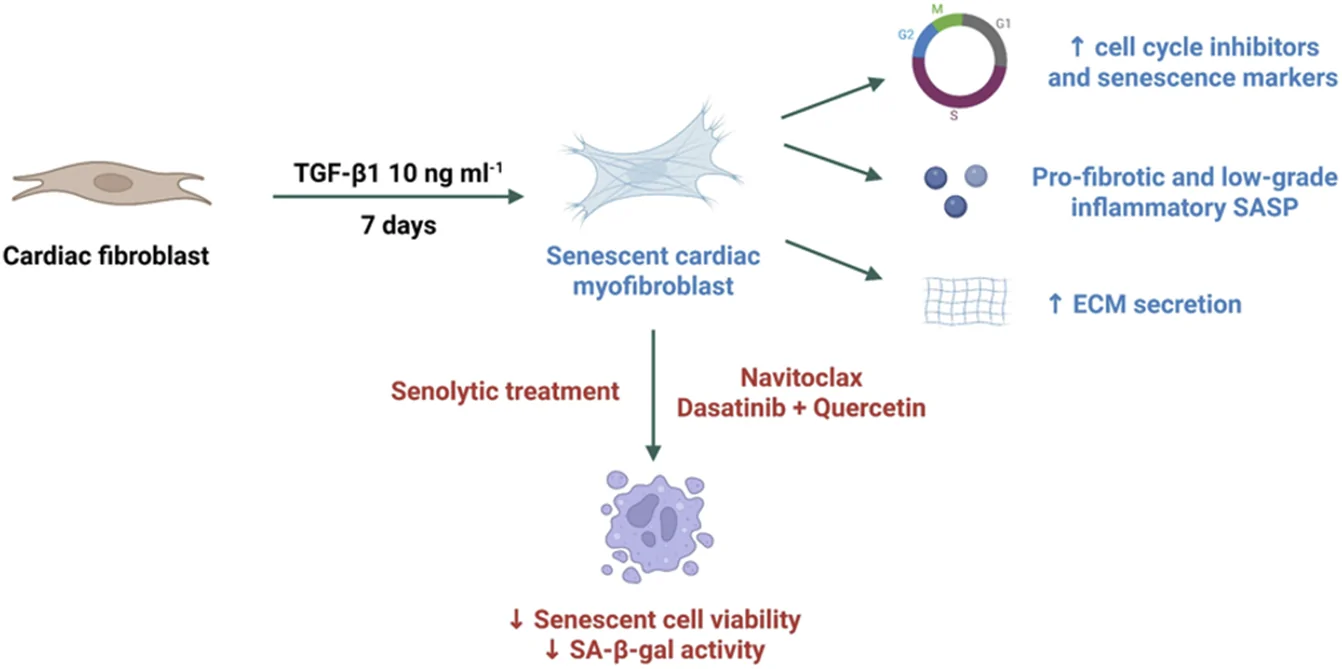
TGF-β1 induces the differentiation of cardiac fibroblasts (CFs) into senescent cardiac myofibroblasts (CMFs). Senescent CMFs produce more collagen, display a low pro-inflammatory but high pro-apoptotic profile, and are selectively vulnerable to Navitoclax and Dasatinib + Quercetin, which reduce both cell viability and senescence markers.

In summary, TGF-β1 drives both differentiation and senescence in CFs, generating senescent CMFs with a profibrotic and low-grade inflammatory phenotype. These cells exhibit increased collagen secretion, altered cytokine release, and a proapoptotic shift associated with Bax upregulation, while maintaining survival through Bcl-2/Bcl-XL expression. Importantly, they are selectively vulnerable to Navitoclax and Dasatinib + Quercetin, which reduce viability and senescence markers (Figure 7). These findings provide mechanistic insight into how senescent CMFs contribute to fibrosis and highlight senotherapeutics strategies as promising therapeutic avenues in cardiac remodeling.

## Data Availability

The datasets presented in this study can be found in online repositories. The names of the repository/repositories and accession number(s) can be found below: Doctoral Thesis of Claudio Espinoza-Perez, Universidad de Chile.
